# The effect of joint mobilization of Maitland on chronic ankle instability: A randomized trial

**DOI:** 10.1097/MD.0000000000039100

**Published:** 2024-08-09

**Authors:** Fang Tang, Shanshan Yin, Pincao Gao, Lin Chen

**Affiliations:** a College of Physical Education, Anqing Normal University, Anqing, China; b Department of Rehabilitation and Healthcare, Hunan University of Medicine, Huaihua, China; c Public Physical Education Department, Taizhou University, Taizhou, China; d Guilin Vocational College of life and health, Guilin, China.

**Keywords:** balance, chronic ankle instability, Maitland therapy

## Abstract

**Background:**

The aim of study was to observe the therapeutic effect of joint mobilization of Maitland on subjects with chronic ankle instability (CAI).

**Methods:**

76 subjects with CAI were recruited for this randomized, single-blinded trial and randomized divided into experimental group (EG) and control group (CG). The CG was received conventional rehabilitation, and the EG added 8-weeks treatment of Maitland technology based on the CG. The visual analogue scale, ankle range of motion, Y-balance test, and Foot and Ankle Ability Measure scores (the daily living part of Foot and Ankle Ability Measure scores and the sport part of Foot and Ankle Ability Measure scores) were measured before and 8 weeks after the intervention respectively.

**Results:**

There was no significant difference on outcomes between the 2 groups before treatment (*P* > .05). After 8 weeks of intervention, the visual analogue scale, ankle range of motion (dorsiflexion, plantar flexion, and varus), the value of Y-balance test (forward extension distance, inner extension distance, and posterior extension distance), the daily living part of Foot and Ankle Ability Measure scores, and the sport part of Foot and Ankle Ability Measure scores of the 2 groups were significantly improved (*P* < .01), and the improvement of the EG showed remarkable than CG (*P* < .01).

**Conclusion:**

Maitland therapy is effective in the treatment of CAI. Conventional rehabilitation assisted by Maitland therapy were beneficial to improve pain and functional state in patients with CAI than only routine rehabilitation.

## 1. Introduction

Ankle sprain is a relatively common sports injury, and about 40% of patients with ankle sprain will suffer from chronic ankle instability (CAI) in the later stage.^[1]^ Despite placing a considerable burden on health and economic sectors, CAI was often dismissed as a minor injury, thought to resolve quickly with simple treatment, therefore up to 30% patients may not receive proper rehabilitation after injury and develop CAI.^[2]^ However, the subsequent symptoms of CAI have adverse health consequences including reduced quality of life and early-onset osteoarthritis.^[3,4]^ Therefore, prompt and effective treatment of CAI is essential.

The pathogenesis of CAI remains unclear, however, patients with CAI have different characteristics, impaired proprioception,^[5,6]^ decreased neuromuscular control,^[7,8]^ decreased range of motion (ROM),^[9,10]^ decreased strength,^[7,11]^ and altered gait^[12]^ are present in patients with CAI. Song et al^[13]^ also have shown that CAI patients are need to compensate for proprioceptive deficits by the presence of more visual information afferents. In addition, some scholars believe that the incoming information of proprioception is blocked due to the destruction of the proprioceptors of CAI patients. Meanwhile, and the mechanism of receiving and processing information in the central nervous system is altered, and the output of movement information is abnormal, which is manifested as delayed muscle response, decreased static and dynamic balance ability, and postural control malfunction, and then there are decreases in the strength of the peripheral muscles of the ankle and limitations of the joints’ mobility, and so on.^[14,15]^

Thus conservative rehabilitation programs of treatment of CAI are designed to improve proprioception, strength, ROM, and neuromuscular control.^[16]^ At present, the therapeutic measures of CAI include foot and ankle orthoses, exercise intervention, Kinesio taping, ligament repair, or reconstruction technology, among them, which exercise intervention is more common in CAI treatment. Although there are many rehabilitation approaches for CAI, there is a lack of standardization and the efficacy of some of these CAI treatment method are not significant. Tsikopoulos K et al^[17,18]^ found that external stabilization measures did not improve dynamic balance than the CG. Luan L et al^[18]^ explored that muscle strength training did not improve the SEBT results. Ling W et al^[19]^ found that Kinesio taping can obviously improve the activation of the long peroneal muscle, but it did not obviously improve ankle proprioception, ankle range of movement and dynamic balance of CAI patients. Otherwise, the previous studies on functional exercises are relatively homogeneous. Maitland technique was created by Australian physiotherapists Geoffrey Douglas Maitland and is an important diagnostic technique for the treatment of musculoskeletal system dysfunction. The technique system includes 2 parts: examination assessment and treatment operation, and has been widely recognized and applied by physiotherapists all over the world. Joint mobilization of Maitland is a physiologically based loosening, which is also the mainstream at present. Related literature report of Maitland technology has a good therapeutic effect on the dysfunction of skeletal and muscular diseases such as arthritis and cervical spondylosis,^[20–24]^ but it is rarely mentioned in the treatment of CAI. In view of these, we add the Maitland technique on the basis of conventional strength training to determine whether it have a significant effect on CAI.

## 2. Materials and methods

### 2.1. Study design

This study was a single-blind randomized controlled trial and its design followed the CONSORT 2010 statement.^[25]^ The study was approved by the Ethics Committee of Hunan Medical University [No. 2020 (H09120)] and complied with the Declaration of Helsinki.

### 2.2. Subjects

Subjects with CAI were selected from the students of Guilin Vocational College of life and health from October 2021 to April 2022.

Inclusion criteria: (1) the subjects met the population screening criteria recommended by the International Foot and Ankle Federation^[26]^; (2) a history of at least 1 significant ankle sprain; (3) have had “loss of control” and/or repeated sprains and/or “feelings of instability”; (4) subjects signed informed consent and voluntarily accepted this trial.

Exclusion criteria: (1) subjects with lower extremity operation history; (2) lower extremity fracture history; (3) other injuries: acute musculoskeletal injuries to other joints of the lower extremity affecting joint integrity and function occurred within the past 3 months, and resulted in at least 1 day of inability to carry out weight-bearing physical activities.

**Figure 1.**
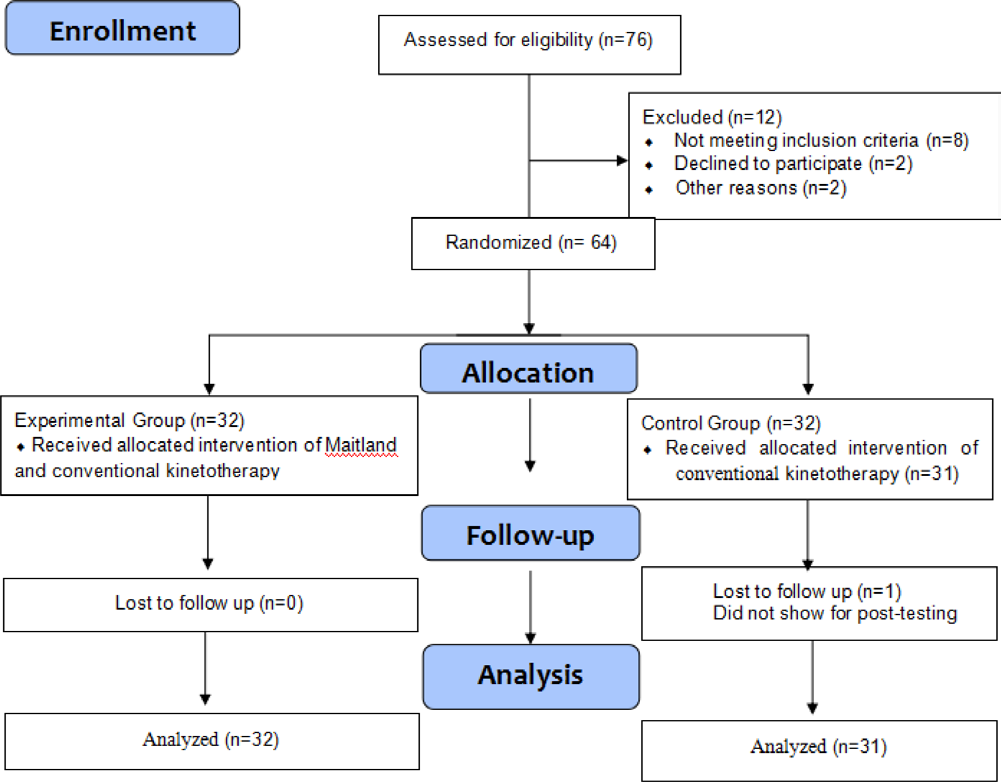
Guidelines flow diagram.

A total of 76 subjects were enrolled for this study. However, 8 subjects did not meet the inclusion criteria, 2 subjects declined to participate in the trial, and 2 subjects were not able to participate in the study for other reasons. Thus, 64 participants were included in the study and randomly divided into the experimental group (EG = 32), the control group (CG = 32) by using a simple random assignment sequence generated by Stata 16.0 software (StataCorp, TX). However, 1 subject was not show for post-tested for other reason in the CG, so remaining 63 participants (EG = 32, CG = 31) completed the intervention. The Guidelines Flow Diagram was shown in Figure 1. The study indicated that there were no significant differences in gender, age, height, weight, course of disease, and affected side between the 2 groups (*P* > .05), which was comparable (Table 1).

**Table 1:** The basic information of subjects.

Group	N	Years	Height (cm)	Weight (kg)	Gender		Injured side		Disease duration (months)
					Man	Woman	Left	Right	
EG	32	28.69 ± 3.57	171.66 ± 6.16	63.33 ± 5.25	18	14	12	20	3.47 ± 0.92
CG	31	27.23 ± 3.38	170.19 ± 5.78	65.46 ± 6.46	16	15	14	17	3.52 ± 0.81
t/χ2		1.667	0.972	−1.442	0.136		0.381		−0.217
*P*		0.101	0.335	0.154	0.454		0.359		0.829

### 2.3. Intervention methods

Prior to the intervention, slips of paper written with the intervention were placed in sealed opaque envelopes by an independent researcher (who was not involved in the intervention) in a 1:1 ratio, and subjects randomly open the envelopes to determine which intervention they received. For CG, conventional rehabilitation training for the ankle was conducted. For EG, routine rehabilitation training combined with Maitland mobilization for the ankle was conducted. Subjects were demanded not to discuss the details of the intervention with the researchers. The above treatment was carried out by a physiotherapist with more than 4 years of experience.

#### 2.3.1. Routine rehabilitation

Subjects in the EG and CG all received routine rehabilitation: kinetotherapy (muscle strength training). Before the training, the subjects performed 5 minutes warm-up activities and 5 minutes internal and external ankle rotation, plantar flexion and extension, and back extension activities. Next, the muscle strength training around the ankle is carried out. The resistance training of ankle dorsiflexion, plantar flexion, varus, and valgus is performed with the resistance band. As the strength of the muscles around the ankle increases, the resistance of the band gradually increases. The form of muscle contraction is centripetal/centrifugal. The subjects were instructed to complete the resistance movements of dorsiflexion, plantar flexion, and varus with maximum strength, 10 times in each group, a total of 5 groups, with a rest of 10 seconds between groups, 3 times a week, for 8 consecutive weeks. The experimental group was supplemented with Maitland therapy base routine kinetotherapy.

#### 2.3.2. Maitland technique

The Maitland technique for CAI includes the following components: (1) longitudinal traction of subtalar joint: subject is supine with heel placed at treatment bedside. The physiotherapist performs a grade III traction with the bone against the long axis of the distal leg. (2) Forward/backward sliding of the subtalar joint: subject is in a supine position with the distal posterior end of the heel, and the physiotherapist then performs a grade III sliding movement of the calcaneus against the talus forward/backward. (3) Subtalar joint internal/external sliding: subject lies on his stomach or on his side, with a towel rolled to hold the ankle joint at the treatment bedside. The physical therapist stabilizes the subject’s talus with 1 hand, then places the palm of the other hand on the inside of the calcaneus, and then performs a level III lateral slide. Or the physical therapist places the other palm on the outside of the calcaneus and performs a grade III medial slide. Training parameters: 30 seconds for each group, 1 minute rest between groups, a total of 3 groups, 10 minutes shared time.

### 2.4. Outcomes

#### 2.4.1. Visual analogue scale was used to assess the pain of the patients with CAI

When measuring, draw a 10 cm horizontal line on the paper, 1 end of the horizontal line is 0 cm, meaning “completely painless”, and the other end is 10 cm, meaning “pain to the extreme”. The pain degree increases progressively from 0 cm to 10 cm, and the higher the score, the more severe the pain degree.

#### 2.4.2. Ankle ROM was used to assess the function of the patients with CAI

The maximum angle of active dorsal extension, plantar flexion, varus of the subject’s ankle is measured using the joint motion measuring scale. Repeat the measurement 3 times and take the average value. The greater the ROM of the ankle within the normal range, the better the function of the ankle.^[27]^

#### 2.4.3. The Y-balance test was mainly used to dynamically evaluate the subjects’ ankle joint balance ability and motor control ability^[28]^

Before the formal test, the subject should make correct demonstration actions. During the test, the subject should stand at the designated point with the affected foot and extend the other foot to the front, back and outward, and back and inward as far as possible. Mark and measure the foot maximum distance for the tip to touch the ground, all values accurate to 0.1 cm. To ensure the accuracy of the Y-test data, we tested the data 3 times for each index and took the average value to reduce its error. The greater the distance, the better the participants’ ankle function.

#### 2.4.4. Foot and Ankle Ability Measure scale (FAAM) was mainly used for screening and efficacy evaluation of patients with CAI

The FAAM scale includes 2 parts: activities of daily living part (FAAM-ADL) and sport part (FAAM-SPORT). The daily activity part has 21 questions in total, with a total score of 84. The physical activity section has a total of 32 points for 8 questions. A higher score indicates better ankle function.^[29]^

Visual analogue scale, ankle ROM test, Y-balance test, and FAAM-ADL, FAAM-SPORT were evaluated before treatment and after 8 weeks of treatment.

### 2.5. SPSS 26.0 software

SPSS 26.0 software (SPSS Inc., Chicago, IL) was used for statistical analysis of all data. The Shapiro–Wilk test was used to evaluate the normality of variables. The mean and standard deviation were reported for the descriptive analysis of the quantitative variables. Independent sample *t* test was performed for comparison before and after treatment between groups, and paired *t* test was performed for comparison within groups. The data of gender and ankle injury side were counted and compared by Pearson χ2 test. *P*-values < .05 indicates that the difference is significant.

## 3. Result

### 3.1. Visual analogue scale

There were no significant differences in the visual analogue scale before treatment between the 2 groups (*P* > .05) in the results. However, after 8 weeks’ treatment, Visual analogue scale was significantly improved in 2 groups compared with that before (*P* < .05), and the improvement of the EG was better than CG (*P* < .05) (Table 2).

**Table 2:** Comparison of Visual Analogue Scale between the 2 groups.

Group	N	Pretreatment	Post-treatment	*t*	*P*
EG	32	4.69 ± 0.97	2.44 ± 0.91*†	9.664	< 0.001
CG	31	4.87 ± 0.82	2.94 ± 0.85*	12.179	< 0.001
*t*		−0.669	−2.234		
*P*		0.506	0.029		

Compared with the same group before treatment,

* *P* < 0.05.

Compared with control group after treatment,

† *P* < 0.05.

### 3.2. ROM

**Figure 2.**
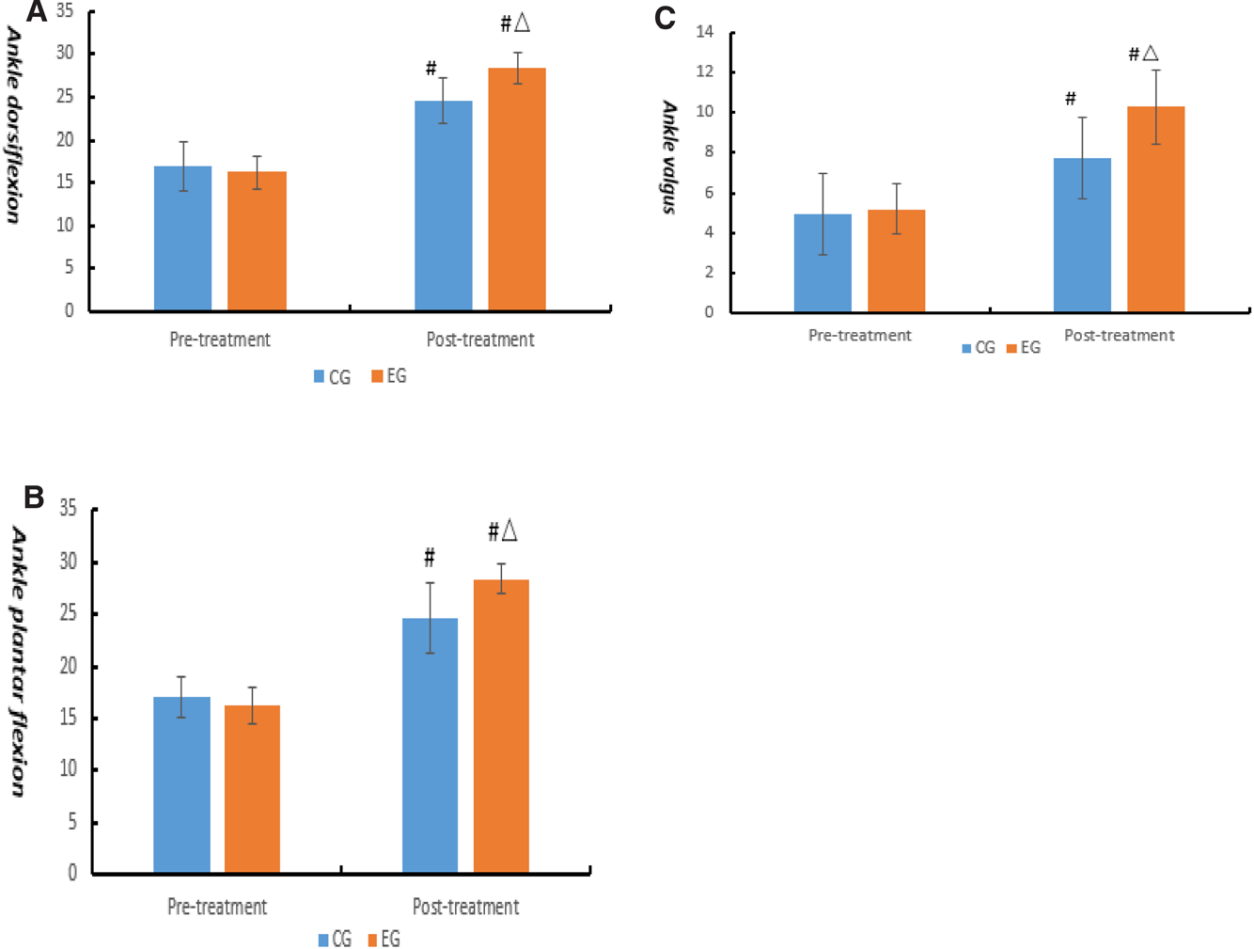
Comparison of ankle range of motion (°) between the 2 groups. Compared with the same group before treatment, #*P* < .05; compared with control group after treatment, △*P* < .05. (A) Ankle dorsiflexion; (B) Ankle plantar flexion; (C) Ankle valgus.

In the term of ankle ROM, our results indicated that there were no significant differences on ankle ROM between the 2 groups before treatment (*P* > .05). After 8 weeks’ treatment, ROM of ankle dorsiflexion, ROM of ankle plantar flexion, and ROM of ankle valgus were prominently enhanced in the 2 groups compared with that before (*P* < .05), but the enhancement of the EG was prior than CG (*P* < .05) (Fig. 2).

### 3.3. Y-balance

**Figure 3.**
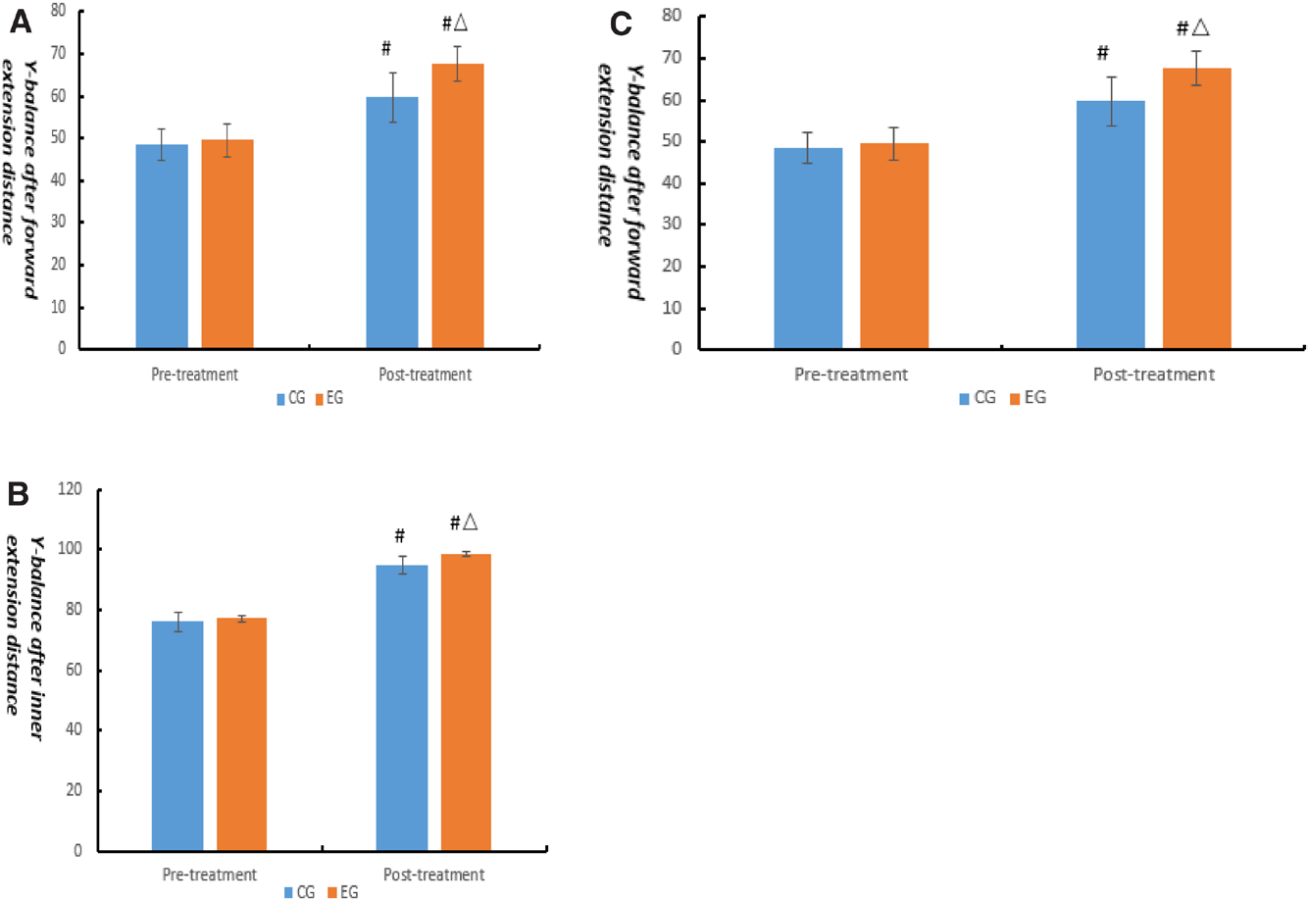
Comparison of Y-balance (cm) between the 2 groups. Compared with the same group before treatment, #*P* < .05; compared with control group after treatment, △*P* < .05. (A) Y-balance after forward extension distance; (B) Y-balance after forward extension distance; (C) Y-balance after posterior extension distance.

The study showed that there were no significant differences on Y-balance between the 2 groups before treatment (*P* > .05). Compared with that before, Y-balance after forward extension distance, Y-balance after inner extension distance, and Y-balance after posterior extension distance were noticeably enhanced in the 2 groups after 8 weeks’ treatment (*P* < .05), and the enhancement of the EG was better than CG (*P* < .05) (Fig. 3).

### 3.4. FAAM

**Figure 4.**
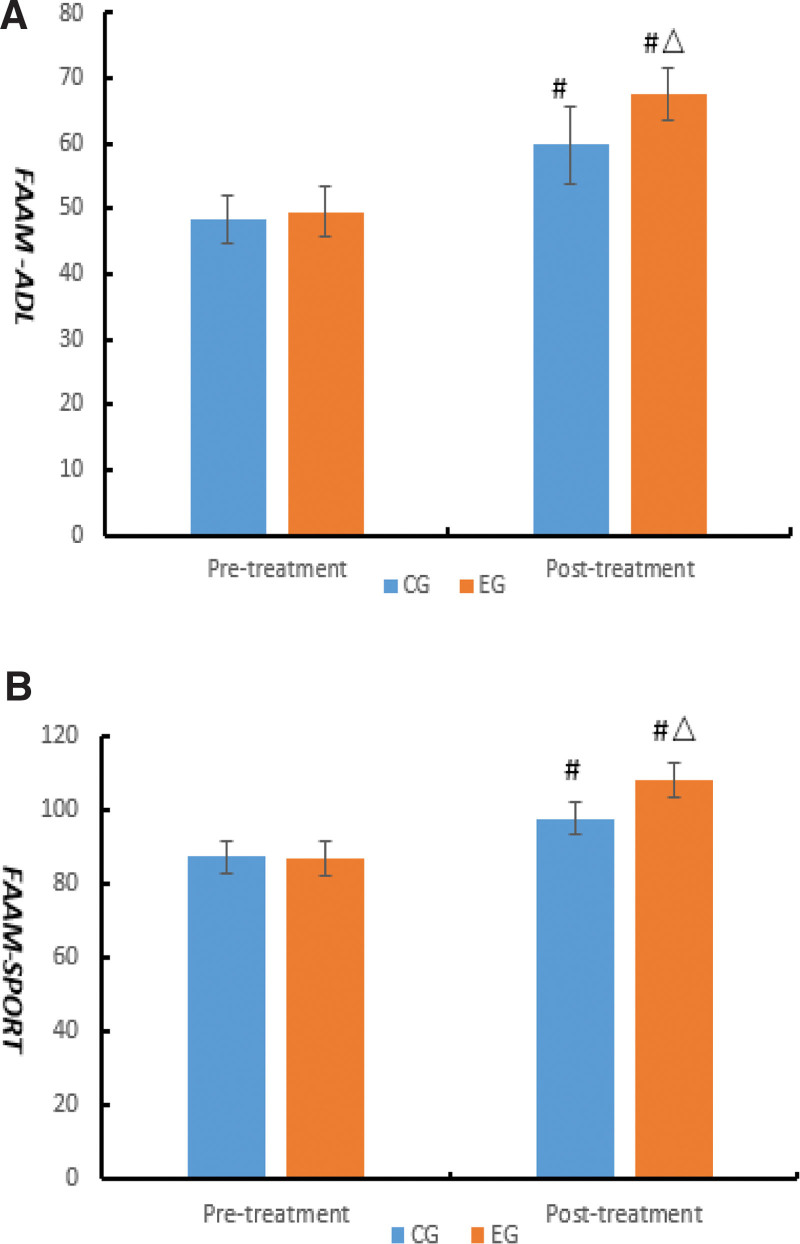
Comparison of FAAM between the 2 groups. Compared with the same group before treatment, #*P* < .05; compared with control group after treatment, △*P* < .05. (A) FAAM-ADL and (B) FAAM-SPORT.

The results indicated that there were no significant differences on FAAM between CG and EG before treatment (*P* > .05). After 8 weeks’ treatment, FAAM-ADL and FAAM-SPORT were noticeably enhanced in the 2 groups compared with that before (*P* < .05), and the improvement of the EG was superior to CG (*P* < .05) (Fig. 4).

## 4. Discussion

Studies have shown that the decline in muscles strength around the ankle, especially the decrease in the centripetal contraction strength of the ankle varus muscle, has a great impact on the reinjury of the ankle joint.^[30–32]^ In addition, the response time of peroneal muscle of the affected ankle was prolonged in patients with chronic joint instability from the perspective of neuromuscular factors, and the muscle strength of varus, valgus and dorsiflexion of the affected ankle joint decreased.^[33,34]^ Therefore, the rehabilitation treatment of CAI should focus on the strength training of muscle groups around the ankle.^[35]^ In this study, resistance training and exercise therapy of joint ROM were carried out on the periankle muscles of patients in both groups. After 8 weeks of intervention, our results showed that ankle joint ROM, Y-balance test, FAAM-ADL and FAAM-SPORT were significantly improved in both groups after treatment compared with before treatment (*P* < .05). It is suggested that conventional exercise therapy such as muscle strength training not only enhance ankle muscles strength but also improve the proprioceptive and balance of the ankle.^[35]^ Otherwise, the magnitudes of the improvement of EG was more noticeable than CG, which suggest that Maitland technology combine routine rehabilitation training may have a prominent role on balance and function of ankle with CAI.

Hubbard et al^[36]^ showed that the distal fibula of most CAI patients presented abnormal forward displacement. This abnormal change in fibula position may result in repeated pulling of the anterior talofibular ligament resulting in altered ligament tension or swelling of the mortise of the ankle joint. During dorsal extension, the talus rotates forward against the calf while sliding backward. Such movement in CAI subjects is limited by the swollen and narrow space of the mortise and leads to the loss of ankle flexibility. The limitation of flexibility may aggravate the mechanical stimulation of the injured lateral ligament. Injury of mechanical receptors leads to abnormal afferent signals and decreased proprioception of the ankle joint. The Maitland technique starts from the “ankle” and deals with the position of the distal and proximal tibiofibular joints to restore ankle flexibility and thus strengthen the proprioception of the ankle to avoid injury. This technique is characterized by a painless dorsal extension of the ankle joint by pushing the external ankle from the back and above while the subject is carrying a heavy load. The state of ankle joint load-closing chain is to simulate the walking situation of the subjects. Normal people have more sliding components of tibia and fibula forward than that of talus backward when walking, and CAI subjects are more likely to have the functional manifestations of ankle joint dorsalis insufficiency under the condition of load-bearing. Due to the limited space for the tibia and fibula to slide forward, the ankle joint is not flexible enough, which leads to the decrease of proprioception. Decreased proprioception can cause ankle joint dysfunction.

Maitland technology can strengthen the intra-articular slip, improve the flexibility of the ankle joint, and increase the sensory output of the receptors in the joint capsule and ligament.^[37]^ A previous study showed that those with CAI have a more constrained sensorimotor system due to numerous sensory and motor impairments.^[38]^ Thus, sensory reinforcement of correct position can further activate motor neurons by using Maitland technology. Other study was found that joint mobilization had a different influence on corticospinal excitability.^[39]^ After the talocrural joint mobilization, the corticospinal excitability of the tibialis anterior muscle was increased. In this study, our results showed that visual analogue scale, ankle joint ROM, Y-balance test, FAAM-ADL, and FAAM-SPORT were significantly improved in EG compare with than CG after 8 weeks’ treatment (*P* < .05). Studies have shown that joint mobilization technique can provide balance and proprioceptive benefits to the ankle joint,^[40,41]^ this may be due to the effects of joint mobilization technique on local articular and neural structures that enhance afferent proprioceptive information from the CAI. This is consistent with the results of the present study. Previous studies have shown that joint mobilization can not only increase the fluctuation of muscle motor potential, but also increase corticospinal excitability, thus contributing to muscle activation. These indicated that Maitland therapy was effective in the treatment of CAI, which has a certain effect on relieving pain, improving ankle joint motion, and promoting movement control and foot and ankle function, and the operation was safe, simple and worthy of promotion.

## 5. Limitations

This study, like others, is not without limitations. For example, a shortcoming of this study is that the participants were not followed for long-term outcomes due to epidemic of the COVID-19.

## 6. Conclusion

In summary, this study suggested that the visual analogue scale, the ROM of ankle, Y-balance, and FAAM of patients with CAI were improved through 8-weeks Maitland technology base on routine rehabilitation, the current Maitland is an effective and reasonably technology that may reduce the ankle reinjury risk in this population.

## Acknowledgments

The authors are grateful to all staff members and all participants who were involved in this study.

## Author contributions

**Conceptualization:** Fang Tang, Pincao Gao.

**Data curation:** Fang Tang, Lin Chen.

**Funding acquisition:** Pincao Gao.

**Methodology:** Shanshan Yin.

**Resources:** Lin Chen.

**Supervision:** Pincao Gao.

**Writing – review & editing:** Shanshan Yin, Pincao Gao.

**Writing – original draft:** Fang Tang.

## References

[1] Gribble PA Delahunt E Bleakley C. Selection criteria for patients with chronic ankle instability in controlled research: a position statement of the International Ankle Consortium. J Orthop Sports Phys Ther. 2013;43:585–91. 23902805 10.2519/jospt.2013.0303

[2] van Rijn RM van Os AG Bernsen RM Luijsterburg PA Koes BW Bierma-Zeinstra SMA. What is the clinical course of acute ankle sprains? A systematic literature review. Am J Med. 2008;121:324–31.e6. 18374692 10.1016/j.amjmed.2007.11.018

[3] Yeung MS Chan KM So CH Yuan WY. An epidemiological survey on ankle sprain. Br J Sports Med. 1994;28:112–6. 7921910 10.1136/bjsm.28.2.112 PMC1332043

[4] Hintermann B Valderrabano V Dereymaeker G Dick W. The HINTEGRA ankle: rationale and short-term results of 122 consecutive ankles. Clin Orthop Relat Res. 2004;424:57–68. 10.1097/01.blo.0000132462.72843.e8 15241144

[5] Willems T Witvrouw E Verstuyft J Vaes P De Clercq D. Proprioception and muscle strength in subjects with a history of ankle sprains and chronic instability. J Athl Train. 2002;37:487–93. 12937572 PMC164382

[6] Forkin DM Koczur C Battle R Newton RA. Evaluation of kinesthetic deficits indicative of balance control in gymnasts with unilateral chronic ankle sprains. J Orthop Sports Phys Ther. 1996;23:245–50. 8775369 10.2519/jospt.1996.23.4.245

[7] Konradsen L Olesen S Hansen HM. Ankle sensorimotor control and eversion strength after acute ankle inversion injuries. Am J Sports Med. 1998;26:72–7. 9474405 10.1177/03635465980260013001

[8] Mckeon PO Ingersoll CD Kerrigan DC Saliba E Bennett BC Hertel J. Balance training improves function and postural control in those with chronic ankle instability. Med Sci Sports Exerc. 2008;40:1810–9. 18799992 10.1249/MSS.0b013e31817e0f92

[9] Drewes LK Mckeon PO Kerrigan DC Hertel J. Dorsiflexion deficit during jogging with chronic ankle instability. J Sci Med Sport. 2009;12:685–7. 18835218 10.1016/j.jsams.2008.07.003

[10] Denegar CR Hertel J Fonseca J. The effect of lateral ankle sprain on dorsiflexion range of motion, posterior talar glide, and joint laxity. J Orthop Sports Phys Ther. 2002;32:166–73. 11949665 10.2519/jospt.2002.32.4.166

[11] Munn J Beard DJ Refshauge KM Lee RYW. Eccentric muscle strength in functional ankle instability. Med Sci Sports Exerc. 2003;35:245–50. 12569212 10.1249/01.MSS.0000048724.74659.9F

[12] Drewes LK Mckeon PO Paolini G. Altered ankle kinematics and shank-rear-foot coupling in those with chronic ankle instability. J Sport Rehabil. 2009;18:375–88. 19827501 10.1123/jsr.18.3.375

[13] Song K Burcal CJ Hertel J Wikstrom EA. Increased visual use in chronic ankle instability: a meta-analysis. Med Sci Sports Exerc. 2016;48:2046–56. 27635773 10.1249/MSS.0000000000000992

[14] Hoch MC Staton GS Medina Mckeon JM Mattacola CG McKeon PO. Dorsiflexion and dynamic postural control deficits are present in those with chronic ankle instability. J Sci Med Sport. 2012;15:574–9. 22575498 10.1016/j.jsams.2012.02.009

[15] Thompson C Schabrun S Romero R Bialocerkowski A van Dieen J Marshall P. Factors contributing to chronic ankle instability: a systematic review and meta-analysis of systematic reviews. Sports Med. 2018;48:189–205. 28887759 10.1007/s40279-017-0781-4

[16] Donovan L Hertel J. A new paradigm for rehabilitation of patients with chronic ankle instability. Phys Sportsmed. 2012;40:41–51. 10.3810/psm.2012.11.1987 23306414

[17] Tsikopoulos K Sidiropoulos K Kitridis D Cain Atc SM Metaxiotis D Ali A. Do external supports improve dynamic balance in patients with chronic ankle instability? A network meta-analysis. Clin Orthop Relat Res. 2020;478:359–77. 31625960 10.1097/CORR.0000000000000946 PMC7438122

[18] Luan L Adams R Witchalls J Ganderton C Han J. Does strength training for chronic ankle instability improve balance and patient-reported outcomes and by clinically detectable amounts? A systematic review and meta-analysis. Phys Ther. 2021;101:pzab046. 33517464 10.1093/ptj/pzab046

[19] Wang L Chen P Wang G Zheng C. Effects of kinesio taping on chronic ankle instability: a systematic review and Meta-analysis. Chin J Tissue Eng Res. 2022;27:2283–90.

[20] Xie L Huang X Yue X Xiao F Han X. Observation on the efficacy of Mulligan dynamic joint release combined with distraction therapy in the treatment of frozen shoulder[J]. Chin J Rehabil Med. 2015;30:476–8.

[21] Wang Y Wang C Chen H Ye X. Electroacupuncture with Mulligan dynamic joint release for the treatment of shoulder pain after rotator cuff injury: a randomized controlled study[J]. Chin Acupunct. 2018;38:17–21. 10.13703/j.0255-2930.2018.01.004 29354931

[22] Zhang J Zhang S Hua X. Efficacy of different treatment frequencies on dynamic joint release for the treatment of lateral epicondylitis of the humerus[J]. J Guangzhou Med Coll. 2014;42:13–7.

[23] Wang Xueqiang ZJ Xu Z. Effect of dynamic joint loosening on secondary frozen shoulder joint motion: a report of 2 cases. Chin J Rehabil Med. 2012;27:358–60.

[24] Reyhan AC Sindel D Dereli EE. The effects of Mulligan’s mobilization with movement technique in patients with lateral epicondylitis. J Back Musculoskelet Rehabil. 2020;33:99–107. 31104005 10.3233/BMR-181135

[25] Schulz KF Altman DG Moher D; CONSORT Group. CONSORT 2010 statement: updated guidelines for reporting parallel group randomized trials. BMJ. 2010;340:c332. 20410783 10.1097/AOG.0b013e3181d9d421

[26] Yu Yue RB. Research progress on screening and classification criteria of chronic ankle instability. Sports Res. 2018;39:94–8.

[27] Tao Q Dong W Sun X Li Y. Efficacy of passive stretching and isometric exercises on ankle dysfunction[J]. China Rehabilitation. 1999:90–1.

[28] Shaffer SW Teyhen DS Lorenson CL. Y-balance test: a reliability study involving multiple raters. Mil Med. 2013;178:1264–70. 24183777 10.7205/MILMED-D-13-00222

[29] Gu L Zhou M Chen Y. Factors affecting proprioceptive recovery after anterior cruciate ligament reconstruction[J]. Chin J Rehabil Med. 2007:1095–6. 19080321

[30] Sefton JM Yarar C Hicks-Little CA Berry JW Cordova ML. Six weeks of balance training improves sensorimotor function in individuals with chronic ankle instability. J Orthop Sports Phys Ther. 2011;41:81–9. 21169716 10.2519/jospt.2011.3365

[31] Yan Y Yang J Chen L Lu H. Rehabilitation effect of proprioception combined with intramuscular effect patch on chronic ankle instability[J]. Hainan Med. 2017;28:2467–9.

[32] Sun M. The effect of PNF technique combined with core stability training on the rehabilitation of functional ankle instability[D] [master’s thesis]. Wuhan Sport University; 2017.

[33] Qin H Pan W Li R Li X. Exercise rehabilitation for chronic ankle instability: current status and characteristics of research[J]. Chin J Tissue Eng Res. 2018;22:5865–71.

[34] Gao Gao CJ Gao J. Changes of ankle muscle strength in patients with chronic ankle instability. J Shandong Univ Phys Educ. 2017;33:53–7.

[35] Hanci E Sekir U Gur H Akova B. Eccentric training improves ankle evertor and dorsiflexor strength and proprioception in functionally unstable ankles. Am J Phys Med Rehabil. 2016;95:448–58. 26745222 10.1097/PHM.0000000000000421

[36] Hubbard TJ Hertel J. Mechanical contributions to chronic lateral ankle instability. Sports Med. 2006;36:263–77. 16526836 10.2165/00007256-200636030-00006

[37] Yan Rubing HC. Progress of clinical application of joint loosening. WestChina Med. 2007;22:917–8.

[38] Wikstrom EA Hubbard-Turner T Mckeon PO. Understanding and treating lateral ankle sprains and their consequences: a constraints-based approach. Sports Med. 2013;43:385–93. 23580392 10.1007/s40279-013-0043-z

[39] Fisher BE Piraino A Lee YY. The effect of velocity of joint mobilization on corticospinal excitability in individuals with a history of ankle sprain. J Orthop Sports Phys Ther. 2016;46:562–70. 27266885 10.2519/jospt.2016.6602

[40] Witchalls JB Waddington G Adams R Blanch P. Chronic ankle instability affects learning rate during repeated proprioception testing. Phys Ther Sport. 2014;15:106–11. 23954386 10.1016/j.ptsp.2013.04.002

[41] Kinzey SJ Armstrong CW. The reliability of the star-excursion test in assessing dynamic balance. J Orthop Sports Phys Ther. 1998;27:356–60. 9580895 10.2519/jospt.1998.27.5.356

